# Supplementary material for: High-Altitude Stress Orchestrates mRNA Expression and Alternative Splicing of Ovarian Follicle Development Genes in Tibetan Sheep
Source: Animals (Basel). 2022 Oct 18;12(20):2812. doi: 10.3390/ani12202812 (PMC9597790; doi:10.3390/ani12202812)
Supplement: Supplementary file 1 [file animals-12-02812-s001.zip › Supplementary Figures S1 and S2.pptx]

## Slide 1
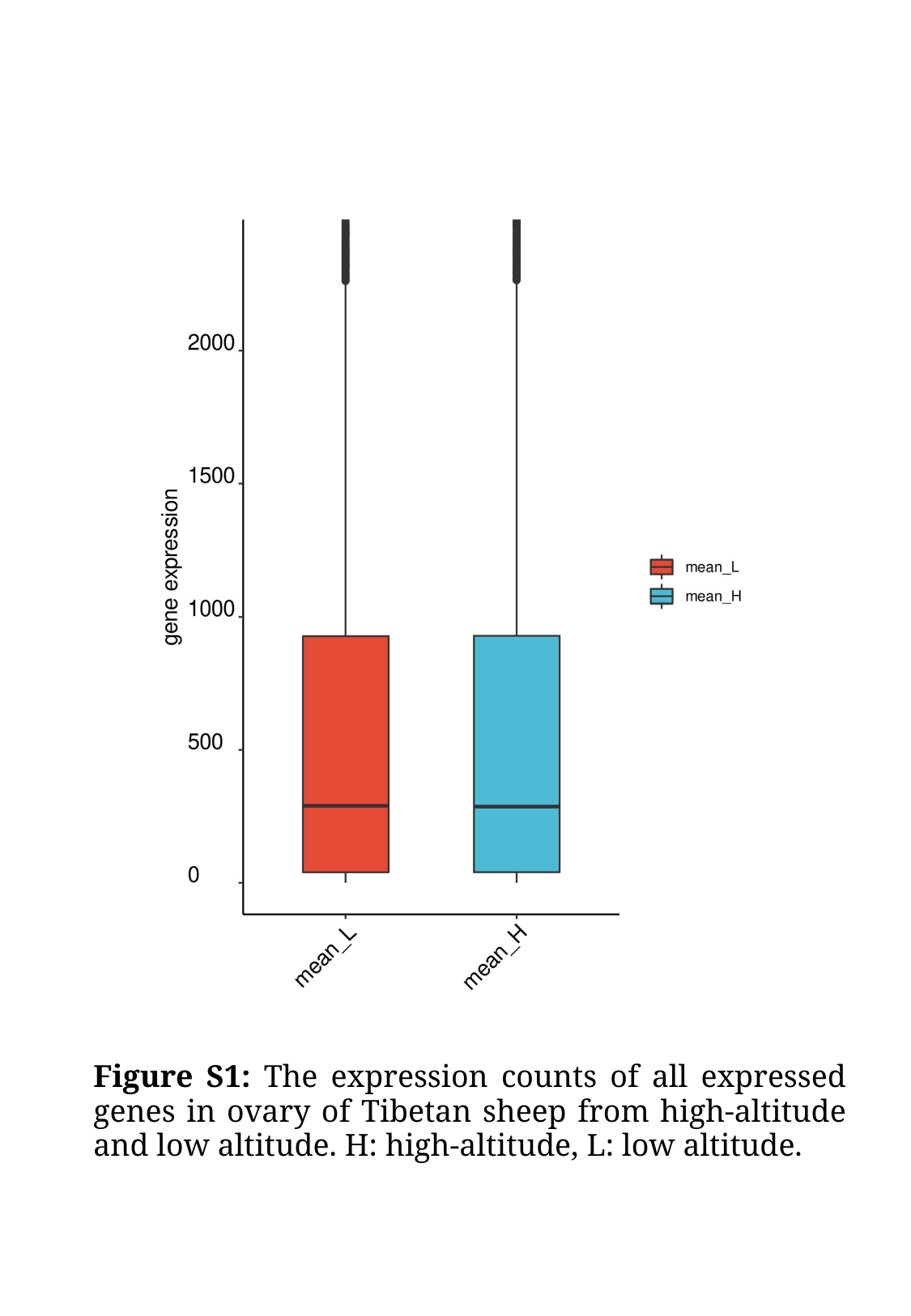

Figure S1: The expression counts of all expressed genes in ovary of Tibetan sheep from high-altitude and low altitude. H: high-altitude, L: low altitude.

## Slide 2
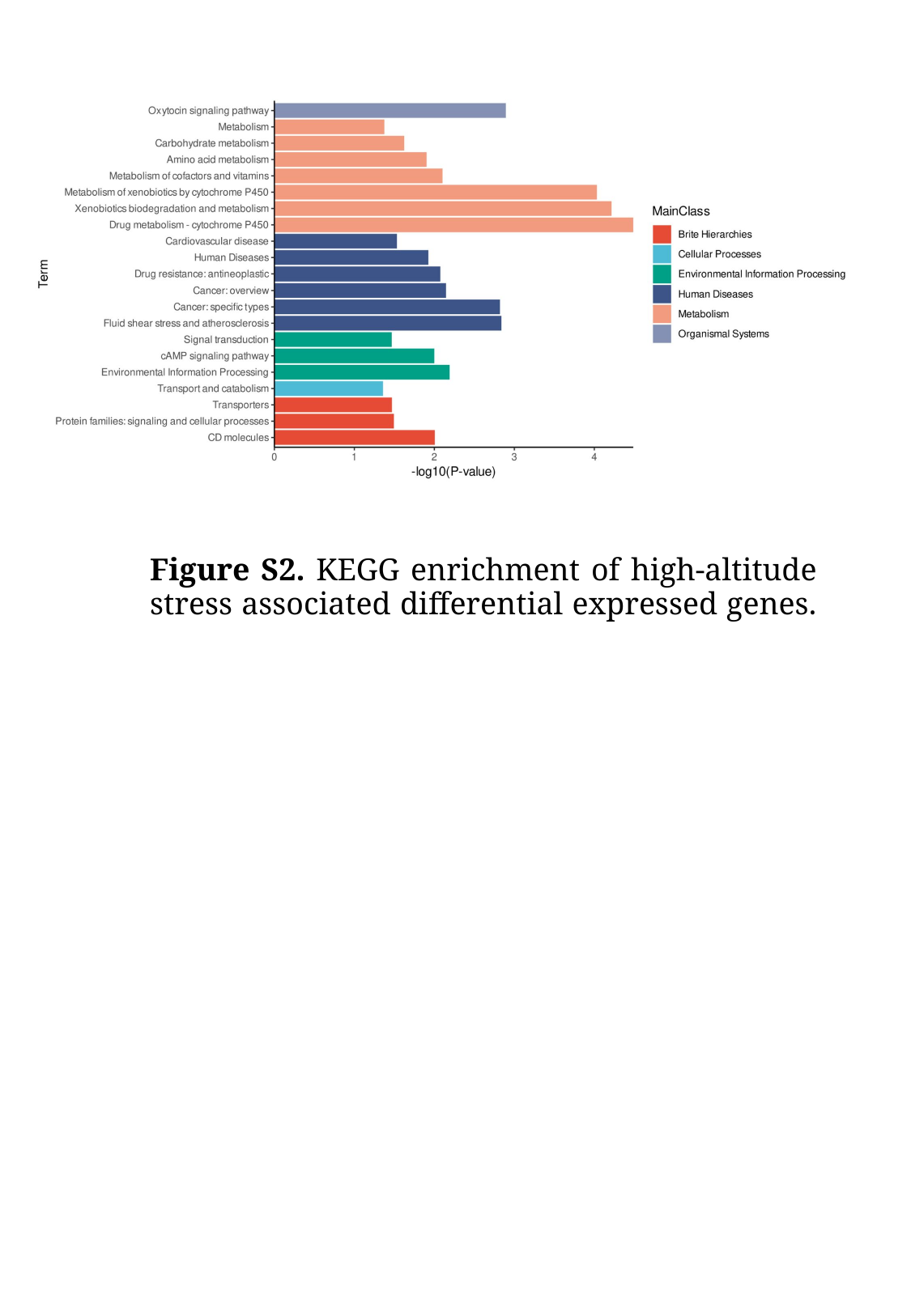

Figure S2. KEGG enrichment of high-altitude stress associated differential expressed genes.
